# Community response to intermittent preventive treatment of malaria in infants (IPTi) delivered through the expanded programme of immunization in five African settings

**DOI:** 10.1186/1475-2875-8-191

**Published:** 2009-08-10

**Authors:** Marjolein Gysels, Christopher Pell, Don P Mathanga, Philip Adongo, Frank Odhiambo, Roly Gosling, Patricia Akweongo, Rose Mwangi, George Okello, Peter Mangesho, Lawrence Slutsker, Peter G Kremsner, Martin P Grobusch, Mary J Hamel, Robert D Newman, Robert Pool

**Affiliations:** 1 Centre for International Health Research (CRESIB), University of Barcelona, Spain; 2 Malaria Alert Centre, College of Medicine, Blantyre, Malawi; 3 Navrongo Health Research Centre, Navrongo, Ghana; 4 Kenya Medical Research Institute, Kisumu, Kenya; 5 London School of Hygiene and Tropical Medicine, London, UK; 6 Joint Malaria Program, Kilimanjaro Christian Medical Centre, Moshi, Tanzania; 7 National Institute for Medical Research-Amani Research Centre, Muheza, Tanzania; 8 Malaria Branch, Centers for Disease Control and Prevention, Atlanta, GA, USA; 9 Medical Research Unit, Albert Schweitzer Hospital, Lambaréné, Gabon; 10 Institute of Tropical Medicine, University of Tübingen, Tübengen, Germany; 11 Infectious Diseases Unit, Division of Clinical Microbiology and Infectious Diseases, National Health Laboratory Service and School of Pathology, Faculty of Health Sciences, University of the Witwatersrand, Johannesburg, South Africa

## Abstract

**Background:**

IPTi delivered through EPI has been shown to reduce the incidence of clinical malaria by 20–59%. However, new health interventions can only be effective if they are also socially and culturally acceptable. It is also crucial to ensure that attitudes to IPTi do not negatively influence attitudes to and uptake of immunization, or that people do not misunderstand IPTi as immunization against malaria and neglect other preventive measures or delay treatment seeking.

**Methods:**

These issues were studied in five African countries in the context of clinical trials and implementation studies of IPTi. Mixed methods were used, including structured questionnaires (1,296), semi-structured interviews (168), in-depth interviews (748) and focus group discussions (95) with mothers, fathers, health workers, community members, opinion leaders, and traditional healers. Participant observation was also carried out in the clinics.

**Results:**

IPTi was widely acceptable because it resonated with existing traditional preventive practices and a general concern about infant health and good motherhood. It also fit neatly within already widely accepted routine vaccination. Acceptance and adherence were further facilitated by the hierarchical relationship between health staff and mothers and by the fact that clinic attendance had a social function for women beyond acquiring health care. Type of drug and regimen were important, with newer drugs being seen as more effective, but potentially also more dangerous. Single dose infant formulations delivered in the clinic seem to be the most likely to be both acceptable and adhered to. There was little evidence that IPTi per se had a negative impact on attitudes to EPI or that it had any affect on EPI adherence. There was also little evidence of IPTi having a negative impact on health seeking for infants with febrile illness or existing preventive practices.

**Conclusion:**

IPTi is generally acceptable across a wide range of settings in Africa and involving different drugs and regimens, though there is a strong preference for a single dose infant formulation. IPTi does not appear to have any negative effect on attitudes to EPI, and it is not interpreted as immunization against malaria.

## Background

Intermittent preventive treatment (IPT) of malaria involves the administration of treatment doses of an anti-malarial drug at predetermined intervals, regardless of parasitaemia or symptoms. Compared to continuous chemoprophylaxis, IPT reduces the number of times an individual has to be given the anti-malarial, and can avoid the problem of delivery if it is given at routine health visits: IPT during pregnancy (IPTp) is linked to ongoing routine antenatal care and IPT for infants (IPTi) is delivered through the Expanded Programme of Immunization (EPI) [1].

Various studies in Africa have shown IPTi with sulphadoxine-pyrimethamine (SP) given at the time of routine vaccinations in the first year of life reduces the incidence of clinical malaria by between 20% and 59% [2-7], and by 30% across the six sites [8]. However, new preventive health interventions such as IPTi can only be considered completely effective if they are also socially and culturally acceptable, and are widely adhered to in the longer-term. It is also crucial to ensure that new additions to EPI do not negatively influence people's attitudes to and uptake of immunization, or that people do not misunderstand IPTi as immunization against malaria and as a result neglect other preventive measures or delay treatment seeking. The importance of acceptability was emphasized in a recent report by the Institute of Medicine [9].

To date the results of two studies that have examined the reception of IPTi, both under the auspices of the IPTi Consortium [10], have been published [11,12]. Because these studies were carried out in the same south-east African coastal region (in Mozambique and southern Tanzania), and both involved only SP, they do not, by themselves, allow the sort of generalization necessary to make wider policy recommendations relating to the acceptability of IPTi across Africa, or for the development of general continent-wide implementation guidelines. They also do not provide any data on the reception of drugs other than SP. There was therefore a need for further research across a range of geographical, political and cultural settings, and transmission areas, and involving a variety of drugs and regimens, so that conclusions applicable to a wide range of IPTi implementation settings throughout Africa could be drawn. Consequently, the IPTi Consortium Acceptability Working Group (AWG) developed a programme of anthropological research that expanded the results from the Mozambique and southern Tanzania studies by using the same approach and methods to examine the socio-cultural issues related to the reception of IPTi in five new settings across Africa. These anthropological studies were all linked to efficacy trials of IPTi (in Gabon, Kenya and north-eastern Tanzania) or implementation studies (in Malawi and Ghana) and involved very different settings and different drugs and regimens (Table 1). The earlier two studies in Mozambique and southern Tanzania had been linked to an efficacy trial [4] and a community effectiveness study [13] respectively.

**Table 1:** AWG study sites and related IPTi Consortium projects

**Site**	**Study design**	**Drug and regimens**
**Kenya**, Asembo (Rarieda district)	Randomized controlled trial	SP + 3 days of artesunate (AS) 3 days of amodiaquine (AQ)+AS, 3 days of chlorproguanil-dapsone (CD), or 3 days of placebo; all arms with daily iron supplementation from 2 to 6.5 months of age Day 1 dose of study drugs provided at 10, and 14 weeks and 9 months EPI visit. On day 2 and 3 the drug was administered in the home under supervision by compliance monitors. Follow-up to 24 months
		
**North-eastern Tanzania**, Korogwe and Same districts		SP Mefloquine (MQ) 3 days of CD, or 3 days of placebo. The drugs were administered at 3, 4 and 9 months, with the first dose of the drug administered under supervision at the clinic and the 2^nd ^and 3rd dose at home unsupervised. Follow-up until 24 months.
		
**Gabon**, Lambaréné		SP or placebo was administered at 3, 9, and 15 months, follow-up until 30 months of age.

**Ghana **(Navrongo), Bawku, Kassena-Nankana, Bolgatanga and Builsa districts	Implementation study	SP at the time of PENTA 2, PENTA 3 and measles vaccinations administered at 2 months, 3 months and 9 months respectively.
		
**Malawi**, Lilongwe and Salima		SP was administered at the time of DPT2 (10 weeks), DPT3 (14 weeks) and measles vaccinations (9 months).

SP – Sulphadoxine-Pyrimethamine

AS – Artesunate

AQ – Amodiaquine

CD – Chlorproguanil-Dapsone

MQ – Mefloquine

DPT – Diphtheria, Pertussis, Tetanus

PENTA – DPT, HepB, Hib

Although this paper focuses on the results of the newer studies, reference will be made to the two older projects as many of the findings are remarkably similar and because the objectives, approach and methods have been so similar as to justify considering the data from all the projects as a single set. However, differences in the design of questionnaires and topic guides between the newer and the older studies have made it impractical to combine the data into a single database. This programme of research is the most comprehensive study of the acceptability and reception of a public health intervention that has ever been carried out.

The scientific objectives of the programme were:

1. To describe knowledge, perceptions, experiences and responses relating to IPTi and EPI of trial participants, community members, and local health care providers across a range of geographical and cultural settings and transmission areas, and involving a range of anti-malarial drugs and regimens.

2. To identify and understand the mutual interactions between perceptions of, attitudes to and experiences with EPI and IPTi.

3. To identify and understand local barriers to the acceptance of and long-term adherence to IPTi.

4. To identify wider socio-cultural, national and regional factors that affect, or may affect, the implementation or acceptability of IPTi.

## Methods

### Settings

The data on which this paper is based come mainly from Kenya, north-eastern Tanzania, Gabon, Ghana and Malawi; countries in which some of the more recent IPTi clinical trials and implementation studies have been carried out. The study in Kenya took place in western Kenya (Asembo, Rarieda district). In north-eastern Tanzania the study was carried out in two sites: a moderate transmission site (Korogwe District, Tanga Region) and a neighbouring low transmission site (Same District, Kilimanjaro Region). In Gabon the study was carried out in the Albert Schweitzer Hospital in Lambaréné town in the Moyen Ogoouée province. The implementation study in northern Ghana was undertaken in the Upper East region, in Bawku, Kassena-Nankana, Bolgatanga and Builsa districts. In Malawi, IPTi was delivered in two districts, Lilongwe and Salima, in the central region of the country. Although IPTi was delivered in all static and outreach clinics throughout the two districts, the acceptability study was conducted in two clinics in Lilongwe and one clinic in Salima.

The choice of study sites was determined by a combination of the scientific need to have as broad and representative a range of settings, drugs and regimens as possible and the pragmatic limitations imposed by the location of other IPTi Consortium studies, the willingness of local research institutions to host the anthropological studies and the funds available. The original intention had been to carry out anthropological studies in parallel to the clinical trials in Gabon, Kenya and Tanzania and the implementation of IPTi in Ghana and Malawi. However, the timing of the various Consortium trials was not entirely synchronized and the idea of conducting a cross-site anthropological study only developed after some of the trials were in a relatively advanced stage. As a result the Gabon trial had ended and the trials in Kenya and Tanzania had completed recruitment and were in the follow-up phase by the time the AWG anthropological study was funded and ready to start. It was therefore decided to carry out a smaller retrospective study in Gabon. In Gabon, Kenya and Tanzania it was not possible for the anthropology team to directly observe the implementation of IPTi. In Ghana and Malawi direct observation was possible as IPTi was being implemented concurrently with the anthropological studies.

### Methodologies

The general approach was anthropological, but the design involved the use of integrated mixed-methods.

#### • Anthropology and grounded theory

Starting from the relatively broad objectives of the programme (see above) and questions and topics developed in the previous IPTi acceptability studies, this study used an iterative, cumulative, process of data collection, ongoing analysis and adjustment of instruments to gradually narrow down to the details of very specific topics. This process resulted in the continual development and introduction of new qualitative topic-specific question guides and questionnaires throughout the programme. New data were continually compared to data already collected through a process of triangulation. This process continued until a point of saturation was reached i.e. when no more new information emerged from the data, and no new insights were generated and a conceptually dense account of the topics of interest were developed, with all categories accounted for, variations within them explained and relationships between them established and confirmed in a range of settings [14,15].

#### • Integrated mixed methods

Data were collected using both quantitative and qualitative methods. Based on a mixed-method model developed to study sexual behaviour change [16] and later implemented in a large multi-centre HIV prevention trial [17]. Qualitative and quantitative approaches were not only used in parallel to collect data but also integrated into the same instrument. For example, the structured questionnaires and in-depth interview guides focused on the same topics and the in-depth interview guides also contained summary fields and multiple-choice answers with tick-boxes that enabled some key responses from the open interviews to be coded and quantified during the interview. As a result, data from both instruments could be included in the same quantitative database and the in-depth interviews provided deeper insights and explanations relating directly to the quantitative data.

Main methods (see Table 2 for additional details):

**Table 2:** Study populations and numbers of interviews and focus groups

**Type of respondent**	**Data collection tool^a^**	**Kenya**	**Gabon**	**Tanzania**	**Ghana**	**Malawi**	**Total**
Participant mothers	IDI	75		63	47	57	242^b^
	QNN	122	119	148	159	137	685

Mother drop outs^c^	IDI	8		21	22	42	93

Fathers	SSI	15		33	80	40	168

Health workers	IDI	27		47	26	25	125
	QNN	12		26	35	44	117

Health worker follow-up	IDI	23		47	15	14	99

Volunteer health worker	IDI	NA		NA	23	15	38

Opinion leaders	IDI	7		26	27	20	80

Traditional healers	IDI	2		23	31	15	71

Non-participant mothers	IDI	NA	38+26^d^	NA	NA	NA	64

Informal conversations			76				76

Participant mothers	FGD	0^e^		19	27	18	64

Community members	FGD	11	10		10	10	31

Community members (non-participant mothers)	QNN		263+231^f^				494

^a ^IDI: in-depth interview; QNN: questionnaire; FGD: focus group discussion; SSI: semi-structured interview

^b ^Not all respondents have been included in the data that were analysed quantitatively as data collection tools varied between Gabon and the other sites. Furthermore, some modifications of the data collection tools across the other sites ensured that not all interviews could be entered in the same database. Therefore, for example although 927 participant mothers were interviewed across the five sites, the total of participant mothers referred to in the quantitative data is lower (805). All data were included for qualitative analysis.

^c ^Although aggregated as "drop outs" across the four sites, the exact definition is different in the implementation studies and the clinical trials.

^d ^In Gabon, due to the retrospective nature of data collection carried out by a consultant, a sample of mothers from the local area were interviewed during two periods of fieldwork.

^e ^In Kenya, participant mothers took part in FGDs organized with other non-participant mothers and hence have been classified as community members.

^f ^Two questionnaire surveys were undertaken in Gabon: with mothers in local schools and mothers at the mother and child clinic.

• **Direct observation **of practical situations relating to IPTi or relevant to the wider context of IPTi implementation (for example, observations of the delivery of routine vaccination and IPTi in clinics).

• **Structured interviews **(1,296) with mothers of infants receiving IPTi or routine vaccination, husbands/partners, IPTi trial staff, community members, local opinion leaders, health care providers and traditional healers.

• **Semi-structured interviews **(168) with fathers.

• **In-depth interviews (IDIs) **(748) with a sub-sample of the above.

• **Focus group discussions (FGDs) **(95) with mothers, community members, health staff and local opinion leaders

• **Text analysis **of relevant documents and messages (media reports, government policy documents and health education materials)

Initial sampling was based on convenience, e.g. mothers who were willing to be interviewed were recruited through study clinics. As the study developed theoretical sampling was used: participants were recruited on the basis of the research team's developing understanding of the field and the questions that emerged from the ongoing analysis of already collected data. The numbers of interviews, FGDs, etc were determined by the saturation discussed above. Data were collected using standardized methods across all the sites except Gabon. The Gabon study was much smaller than the others and consisted of two fieldwork periods of two months and one month respectively. Due to the difficulty in recruiting local social scientists for such a short project, consultants were hired to collect the data. In the other sites fieldwork lasted for 18 months and was carried out by a site-based team of three to four interviewers, headed by a site principal investigator (PI) (who was also the local PI of the related Consortium trial or intervention study) and a senior social scientist.

Participants were selected as follows:

• Participant mothers were all selected through the study clinics. In Kenya and Tanzania they were selected during IPTi trial follow-up visits or, after follow-up was completed, during routine clinic visits. In Gabon children who participated in the IPTi trial and their siblings continued to receive free medical treatment after follow-up and mothers were recruited for the acceptability interviews during such visits. In the implementation sites in Ghana and Malawi women were selected from among those attending the EPI clinic. Although interviewers ensured that they selected a representative range of participants with regard to age, selection was also to a large extent by convenience, because they had to fit the interviews into the busy clinic routine and catch women in waiting areas before of after they were attended to. Women who had "more to say" were selected for follow-up interviews.

• Drop-out mothers were identified from clinic data and traced at home.

• Fathers were recruited through mothers who were interviewed and who consented to their partners being approached with a request for an interview.

• All health workers at the study clinics were interviewed.

• Traditional healers were chosen from among those in the vicinity of the clinic who were known to treat infants.

• Opinion leaders were selected based on recommendations from local community members and clinic staff.

The analysis of the qualitative data has been through a Grounded Theory approach [14], with more abstract generalizations emerging from the data based on the coding of emerging themes using Nvivo 2, a computer programme which enables the management, coding and analysis of large sets of qualitative data. Descriptive statistics from the qualitative data were generated using SPSS 14.0. Data were compared across the sites to generate a comparative picture of the reception of IPTi.

### Ethics procedures

Overall ethics clearance for this study was obtained from the Ethics Committee of the Comité Ético de Investigación Clínica of the University of Barcelona. Separate local ethical clearance was obtained at each site, and for Kenya and Tanzania additional clearance was obtained from the London School of Hygiene and Tropical Medicine and the Centers for Disease Control and Prevention, Atlanta. Informed consent was obtained from all study participants in accordance with local ethics committee regulations.

## Results

### Acceptability and adherence

A number of key factors have emerged from the data across all the study sites that influence the acceptability of IPTi in either positive or negative ways. These factors relate to the local cultural and health-care context, the clinic environment and the nature of the drugs and regimens used for IPTi. There is a great deal of similarity across the sites.

#### Local health culture and the routinization of EPI

In all the study communities there was a general and widespread concern about infant health, and an underlying assumption that one should do all that is possible to ensure infant well-being. This is a strong motivation for mothers to comply with prevention practices and programmes, either traditional or biomedical, whether they fully understand them or not. Non-adherence to any activity that is perceived as being beneficial to infant health is likely to be seen as neglect, and so there is strong social pressure to comply and be seen as a "good mother".

More than half of mothers in Tanzania, Kenya, Malawi and Ghana (53% [427/805]) said that traditional practices for preventing illness or ensuring health and well-being in infants were widely used. There was some variation across the sites (Kenya 22% [43/197], Tanzania 54% [112/208], Ghana 62% [127/206], Malawi 75% [145/194]). These practices varied from interventions aimed directly at babies and infants (such as ritual or herbal treatments carried out by traditional healers or the wearing of amulets) to more indirect measures aimed at parents (such as the observation of postpartum sexual abstinence and seclusion by mothers). In Gabon, mothers mentioned traditional immunization practices in 52 (of 64) open interviews. In these interviews 34 women said they had used traditional "vaccination", mainly for their children, but also for themselves. Such traditional practices were also commonly reported in the earlier studies [11,12].

Interviewer: How does it work, what does the traditional healer do?

*Mother: Vaccination*.

Interviewer: Are these vaccinations like scarification, what does the traditional healer do?

*Mother: In fact they vaccinate against certain spirits...it is to prevent all contact with what the sorcerer could do and to prevent the illnesses he could send, so that these don't affect you*.

(IDI, mother, Gabon)

This traditional prevention may involve rituals or herbal treatment.

Most women (87% [700/805]) said that they used bed nets for preventing malaria (Tanzania 98% (204/208), Kenya 99% (195/197), Ghana 84% (173/206) and Malawi 66% (128/194)). Usually nets were distributed free at clinics to mothers who had just given birth or when vaccinating their babies. In Kenya, nets were distributed to participants at the time of enrolment. Across the sites one quarter of mothers (24% 192/805) also said they used other means of (perceived) malaria prevention, such as clearing bushes around their compounds, clearing gutters of standing water, or fumigating their houses using spray or by burning leaves, seeds, or dung. This also varied across the sites, from 50% in Ghana, where insecticide sprays were popular, to 7% in Tanzania. These practices were also frequently described in the FGDs.

Women were generally familiar with, and accepted, IPTp. Women in some of the sites spoke about IPTp and IPTi as a continuum, with IPTp providing protection against malaria for both mother and child before birth and IPTi providing protection for the child after birth. This was reported less often than in the Mozambique and southern Tanzania data [11,12], but was not mentioned at all in Kenya.

EPI was generally well established at the study sites and had largely become routinized. However, knowledge of vaccination was often not detailed. Most mothers [87% (702/805)] knew that vaccination prevents disease (there were minimal differences between the sites), but only 2% (20/805) knew exactly which diseases were prevented (10/20 were from Tanzania). Eleven percent of mothers (88/805) mentioned that vaccination was "the law" which they were obliged to follow (43/88 were from Tanzania, 27/88 from Kenya).

*It is the law that you must take your child to the clinic*.

(IDI, mother, Ghana)

Facilitator: Why do you think women come for vaccinations?

*All: It is a law*.

Facilitator: A law from where?

*Mother: A law from the government*.

Facilitator: What do they do to you if you don't attend the clinic for vaccination; do they send you to the prison?

*Mother: They don't send you to the prison but it is just important*.

(FGD, mothers, Tanzania)

Most women were critical of other women who, they claimed, neglected to have their children vaccinated due to laziness or ignorance.

Although mothers sometimes reported side effects of vaccination, mainly fever (38% [304/805]) local swelling (6% [47/805]) and the infant crying (6% [47/805]), they generally did not consider these to be serious (there was little difference in the profile of side effects reported across the sites, except "crying" was more readily reported in Malawi. There was however variation in the proportion of mothers reporting side effects across the sites: Tanzania 24% [51/208]; Malawi 46% [92/194]; Kenya 48% [95/197]; Ghana 58% [120/206]). Of the mothers who reported side effects, 64% (228/357) said that they were not serious. Some saw these mild side effects as evidence that the vaccination "worked".

*It is true actually even my youngest child got swelling, but I have noticed that after vaccination the child must get fever. When the child gets fever it means the vaccination is working. So we take this as a normal thing*.

(IDI, mother, Tanzania)

However, in Gabon 21% (103/494) of mothers said they knew other women who did not take their infants for vaccination because of the fever resulting from vaccination.

Less commonly reported side effects that were perceived as serious, such as abscesses, did deter a few women from returning to the clinic for the following vaccination. Three percent (23/805) of mothers reported (what they perceived to be) serious side effects (15/23 were from Malawi). Mothers were sometimes concerned when their child received a previously missed vaccination on top of the scheduled one because they thought this might be too strong, and this was also mentioned as a reason for not returning to the clinic.

For many women in the implementation sites in Ghana and Malawi, IPTi was simply an unnoticed addition to an already routinized EPI. However, sometimes it was recognized as something new, and the data from direct observation show that in Ghana some women with babies falling outside the age range did come to the clinics requesting 'the white drug'.

A majority of mothers (88% [709/805]) across the sites reported the experience of IPTi as positive. Only in Malawi did a noticeable number of mothers report their experience as neutral: 15% [29/194]). Some participants compared the IPTi child with their siblings and perceived a difference in the number of malaria episodes between them. In the implementation sites, some mothers thought that IPTi was meant to prevent the child from getting fever after being vaccinated as they thought it was a pain killer.

Fathers in the trial sites in Tanzania and Kenya were more likely to have heard about IPTi (all 48 interviewed fathers) compared to those in the intervention sites in Malawi and Ghana (44% [53/120]). In the trial sites fathers appear to have played a key role in decision-making relating to IPTi because the intervention involved participation in research. The interviews with fathers reveal that women in the non-trial sites tended not to inform their husbands as much about IPTi.

In the study sites fathers are generally expected to provide financial support for health care and are, because of this, the ultimate decision makers regarding health care seeking for children. However, they tend not to play a direct role or be interested in day-to-day infant care (which includes routine health seeking and treatment for minor ailments). Most mothers (57% [455/805]) said that they did need to consult their husbands whether to take their children to the hospital, as there were costs involved, but not for vaccination as it was free (Ghana 81%, Malawi 76%, Tanzania 45%, Kenya 24%). However, as soon as health-related issues are perceived as non-routine (for example, in the case of a clinical trial, or the introduction of new drugs) then fathers do become more interested and often intervene (negatively), based largely on ignorance of the facts and often stimulated by rumours, which they play a part in spreading (based on in-depth interviews and focus groups with mothers, fathers and health workers).

*That is the issue of blood [stealing] that they [fathers] said was the major cause of their kids getting more sick, and others were saying that we were testing HIV/AIDS without their consent while pretending that we are doing a malaria study*.

(IDI, health worker, Kenya)

*Mother: I really longed to join [the IPTi trial] because the child would be treated free of charge, but [the father of the child] had some issues with this and I couldn't force him*.

Interviewer: What was the issue?

*Mother: It was the blood issue. People said that these children were sickly and that they took a lot of blood from them. Because he had heard these things, he didn't understand even when I tried to explain. I even gave him that form [information leaflet] and he read it. He also complained that the drugs that were going to be used were powerful and if the child's body got used to them, he would develop some problems in the future if he completed the study*.

(IDI, trial drop out mother, Kenya)

*People were telling us not to take part in the project because the children will get big heads [hydrocephalus] and they will also get brain disorder. They claimed that white people aimed to test their drugs on our children. Those who didn't understand, they refused to take part in the project*.

(FGD participant, Tanzania)

*Interviewer: You said people mentioned *mumiani *[vampires, blood-stealers], but generally, how did the community respond to the project?*

*Opinion leader: Their response was good. Those who talk about *mumiani *are the minority who do not understand, but the majority accept the project*.

(IDI, opinion leader, Tanzania)

When asked what they would do if their husbands refused to allow them to take their children to the clinic not all mothers responded; they could not imagine or had not experienced such a situation. However, of those who responded, 46% (239/518) would covertly or overtly defy their husband; 11% (55/518) would obey their husband; and other respondents reported that they would try to persuade their husband; they would postpone the question; or were unsure. In explaining their defiance mothers referred to the authority of the government or the responsibility they were expected to assume for their baby's health. The proportion of mothers who would defy or obey their husband varied over the different sites. For example, those who said they would obey ranged from 2% in Malawi to 15% in Ghana, and those who would defy ranged from 30% in Malawi to 68% in Ghana.

#### The culture of the clinic

Health workers appeared to be generally motivated and committed, and to encourage mothers to keep to the routine vaccination schedule and to comply with treatment regimens. This emerged from interviews and focus groups with mothers and community members, and was supported by follow-up interviews with health workers about their motivation for choosing this occupation.

*I had an interest and wanted to become a health worker. I really liked seeing other workers giving out vaccines to babies and helping them to get the best medication and to grow up healthy. Thus I wanted to become one of the people that was helping people and babies to grow up healthy and strong*.

(IDI, health worker, Malawi)

However, health workers also often complained that their effort was not adequately rewarded, and some of those involved in IPTi at the implementation sites felt that they had been inadequately trained (62% [65/105]). Those who had not received IPTi training felt resentful when they were called upon to provide IPTi. Health workers often reported being generally disappointed that patients did not follow their advice.

Most mothers seemed to generally trust and respect health workers, and in in-depth interviews frequently said they had been assisted in a friendly way and they appeared to do their best to conform to health workers' expectations and show that they were good and responsible mothers. Health workers were perceived as providing an essential service.

...because the doctor or the nurse is in a way like Jesus who has come to save us...

(IDI, mother, Gabon)

However, it was clear that when they perceived patients to be non-compliant health workers became frustrated and as a result sometimes publicly reprimanded or ridiculed them. In the in-depth interviews 8% (20/242) of mothers referred explicitly to insults or reprimands from health workers.

*The doctor on duty assesses if the child has been attending EPI, if not, the mother of the child is reprimanded, sometimes they serve her last. But usually, they give a lecture to other mothers about the consequences of not attending EPI, and use the mother who was not attending as an example. The idea is to warn other mothers that they will be embarrassed if they do not attend EPI*.

(Observational field notes, Malawi)

Interviewer: What would have happened if you refused to collect the white drug, and later you went to the clinic?

*Mother: The nurses would insult me because it means I do not want my child's welfare; I do not want to prevent disease*.

(IDI, mother Ghana)

There is even the occasional report of physical punishment:

*One day I took the child to the hospital and when the nurse examined the child she immediately gave me a slap and rebuked me for waiting all this time when I could have brought the child much earlier. She told me that if I had waited another day the child would have died*.

(FGD, mothers, Ghana)

In their interaction with clinic staff women tended to be passive: they usually did not ask questions for fear that this would be interpreted as a sign of ignorance and they would be ridiculed by staff and laughed at by other women, and they accepted this treatment without resisting or questioning it.

*It may be good or not...we just receive it because the doctors say so*.

(FGD, mothers, Malawi)

*We don't know the difference between them [different medications] but we take them because they give them to us and tell us: 'take it' and we just do it*.

(FGD, mothers, Ghana)

This passive acceptance was partly due to a local culture of subservience (the underlying assumption being that one should obey those higher up the social and educational hierarchy) and partly due to fear of being refused help when they might need it later (for example when they come for delivery). Both of these factors were also important in Mozambique and southern Tanzania. Once they have missed one clinic session, mothers may then be less inclined to return to the clinic out of fear that they will be punished or ridiculed.

*Sometimes there is no respect here, and this discourages women, those who don't like to be insulted*.

(IDI, mother, Gabon)

*Also because of the way the nurses insult people who miss clinic in the full view of other mothers, nobody wants to be insulted by them so you will always try to go every time*.

(FGD, mothers, Ghana)

If the culture inside the clinic was based on hierarchy and authority, where the approval of health workers determined access to treatment, there was also a parallel culture outside the clinic, in the waiting spaces where mothers met each other and socialized. At all sites, vaccination clinics were social events to which many women looked forward as an opportunity to dress up and get away from the daily routine of farm work and household chores. As inside the clinic, this more informal social space also formed a context for approval and disapproval – this time from fellow mothers rather than health workers – and while "good" mothers and well-dressed women received approval, those who did not conform were ridiculed and stigmatized (whether actually or in their own estimation). This could also be a source of inequality for mothers who did not have the means to keep up appearances, and could lead to non-attendance.

For example, most of the young mothers interviewed in Gabon (80% [184/231]) claimed that shame (*honte*) related to having sick or malnourished infants, having too many children, or being a young mother was a common reason for avoiding routine clinic visits [18].

Facilitator: Are there some people that do not go to vaccinate their children?

FGD participant: For some it's lack of family planning...having a lot of kids with no child spacing...

Facilitator: So how would that stop someone from taking their kid to get vaccinated?

Participant: The mother feels shy/ashamed, because she has a child on her back and she is expecting another and can't carry the third kid...

Several participants: Yes it's true...another child may be crawling...another learning to sit...

Facilitator: She becomes shy because of who.do the doctors laugh at her at the hospital?

*Participant: Because of the other women too...she stays away from them because she knows that what she did is wrong...child bearing in such a manner*.

(FGD, community members, Malawi)

*Health worker: When we go for outreach [clinic] most mothers don't come, so it's difficult to get them*.

Interviewer: Why don't mothers come for outreach?

*Health worker: It's because most of them prefer to go to the main clinic to meet their colleagues, others too want to come and exhibit their new dresses*.

(IDI, volunteer health worker, Ghana)

*Some of them look shabby...it could be that you have only one piece of clothing that you use everywhere you go, while others come to the clinic gorgeously dressed. So they [health workers] discriminate against you by attending to them first. These things discourage you*.

(FGD, mothers, Ghana. Discussing why some women do not take their children to the clinic)

These cultural factors were exacerbated by (perceived) geographical and structural constraints, such as distance to the clinics, poor infrastructure and long waiting times:

*If I do not get to the clinic on time, they have finished talking to the women. It's because of the distance. When I get up I have to clean, bathe the children and cook first, and all that makes me late so I don't hear anything [of the health education talk]*.

(IDI, mother, Ghana)

#### Drugs, regimens and delivery mode

The most important direct determinants of the acceptability of an intervention are whether people perceive it as doing what they think it should do, and whether the benefits are perceived to outweigh the disadvantages. Side effects (or rather perceived side effects) are an important factor in determining acceptability. In this study 11% [85/805]) of mothers (mostly in Kenya and Tanzania) reported (perceived) side effects of IPTi (44 referred to fever, 30 to vomiting, and 13 to diarrhoea). Only 14 mothers reported what they perceived to be serious side effects from IPTi (all in Tanzania and Kenya), which included diarrhoea (4), chest pain (2) convulsions (1), sickle-cell anaemia (1) and rashes (1); five mothers did not provide specific details. As in the case of vaccination described above, the side effects of IPTi, when they were reported, often tended to be interpreted as evidence of the drug's potency.

Across the sites, the views of study participants on SP were mixed. There were those who liked SP: they were familiar with it, they had used it for years, they knew it was generally safe, in spite of rumours at some sites (derived from newspaper reports) about it causing people's skin to peel off.

*SP is good; I have used it, and my child has used it, and the same applies to my wife when she was pregnant, so it is good*.

(SSI, father, Tanzania)

In Gabon, 71% (85/119) of trial participants said that the drugs given as part of the trial were "good" – 56% (67/199) "knew" that their infants had received SP.

On the other hand, the fact that it has been replaced as first line treatment suggested to some mothers that it was no longer effective, and the newer drugs introduced to replace SP were generally perceived as stronger and more effective. This was especially true in the trial sites where drugs that were being researched were seen as more powerful than those currently being prescribed.

*Mother 1: For me Fansidar [SP] never works. I have always taken quinine. It works better. It is the real medicine for malaria; and aspirin and Panadol*.

*Mother 2: For me too, Fansidar never works for me. I think that quinine is the best. Fansidar does not do anything. It never makes anyone better. Everyone complains about Fansidar nowadays. I think it stopped working. I don't know; is it still the same people who make it? It is not like it used to be*.

(FGD, mothers, Malawi)

The other side of this is that the newer drugs were often perceived as being too strong for people who have a weaker constitution, such as infants and the elderly. The fact, mentioned above, that in the trial settings infants are often reported to have vomited after receiving the trial drugs, was sometimes interpreted as evidence of this.

Both mothers and health workers generally did not like the crushing of tablets (33% [57/174] of health workers said it was "difficult") and their administration to the child using cups and spoons (46% [53/116] of health workers from the implementation sites in Ghana and Malawi described it as impractical). Mothers were mainly concerned about hygiene, but the practical difficulty of getting infants to swallow the drug and the fact that they often spat most of it out again also played a role. Observations in the clinics made it clear that infants often spat out a substantial amount of the drug. There were also concerns in the implementation sites about the administration of water to infants and how this might contradict advice on exclusive breastfeeding.

*I really do not like the way that the Fansidar [SP] is given. We all have to use the same cups. They don't even rinse them out properly, they just throw them into the water and take them out, and that's it...The problem is that we can't complain. If you did, they would turn against you and ask you who you think you are, complaining when no one else is*.

(IDI, mother, Malawi)

In Kenya, both mothers and health workers reported (in interviews and FGDs) that the introduction of a syringe to squirt the dissolved drug into the infant's mouth was a big improvement. However, some mothers did not like the look of the syringe, which made it seem like their child was being injected in the mouth. There was general agreement among both mothers and health staff across the sites that an infant formulation would be necessary to ensure acceptance in the long run.

In Tanzania, the trial included an aspect of home administration. The first of three doses was given at the clinic and the mother administered the remaining doses at home on the following two days. Monitors (12/13) who followed the mothers up to check they had given the subsequent doses claimed that some women did not comply and that they forgot to administer the drugs or kept the tablets for subsequent use.

In Kenya, the trial also included home administration. However there the compliance monitors travelled to the mothers' homes with the drugs and supervised their administration (waiting 30 minutes to ensure that the infant did not vomit). During interviews Kenyan compliance monitors reported being sceptical about mothers' ability to administer drugs without supervision and 4/6 of them referred to cases of non-compliance with trial procedures.

In Tanzania, the monitors had varied opinions about mothers' ability to administer drugs correctly without supervision. Although some were pessimistic, they suggested that the trial context may negatively influence mothers' compliance (due to suspicion about the drugs) and that adherence might be greater in a non-research setting.

Various other factors relating more or less directly to the mode of delivery also impeded the implementation of IPTi. So, for example, in some of the clinics gender roles interfered:

*Obviously I have trouble [delivering IPTi] because, being a man, I can't just take the cups and wash them. I have to leave them because that is something that the women are supposed to do. That means that whoever comes on that day for the IPTi will not receive it*.

(IDI, health worker, Malawi)

While such explicit statements were not common, they did express much broader underlying issues that impinged on the quality of care in these settings.

In the trial sites, the free medical attention provided to infants acted to counter-balance the scattered reports of side effects, beliefs about the drugs being too powerful, and the rumours about blood stealing and covert HIV-testing. The free medical attention was a commonly cited motivating factor for trial participation, particularly in Kenya.

In Malawi, the fact that the measles vaccine came in preset quantities meant that if the number of babies needing vaccination did not match the amount of vaccine in the bottle, the remaining women were sent home without their infant having received either a measles vaccination or IPTi because the health workers were reluctant to waste medicine by opening a new bottle.

In the implementation study sites, particularly in Ghana, there were reports of busy health workers handing the dissolved SP tablets to the mothers and permitting them to administer the drug to their infants. This was also observed in the clinics in Malawi (though when staff were questioned they denied this). This led to some cases of mothers not administering all the drug solution.

Scattered reports also emerged from Malawi and Ghana of health workers providing mothers with the SP tablets and instruction on how to administer the drug at home. This also occurs in the implementation site in southern Tanzania [11]. In Ghana, the health workers apparently allowed more educated mothers to do this, as they were under pressure for resources, particularly the cups used to dissolve the SP tablets.

### IPTi-EPI interactions

There is little evidence in any of the study sites that IPTi per se had a negative impact on attitudes to EPI or that it had any effect on EPI adherence. Only 2% (6/805) of mothers said that IPTi had discouraged them from EPI attendance. Of the fathers 6% (10/168) said that IPTi might be a reason for them to discourage EPI clinic attendance. However, this was related to research procedures, in particular blood taking. Because EPI was generally valued positively, IPTi benefited from being associated with it.

Mothers generally did not think that having received IPTi meant that their child would no longer get malaria. This was not because they were aware of the limited protection actually offered by IPTi, or the fact that at some of the sites the drugs were being tested in efficacy trials, but rather because they generally viewed all prevention practices, whether traditional or biomedical, as partial.

*The disease won't hit him as much as when he would have never taken any medication*.

(IDI, mother, Gabon)

This view of prevention resonated with traditional preventive practices for infants that were common across all the sites (see below). These findings are the same as those from Mozambique and southern Tanzania.

However, across the sites a minority of mothers (16% [126/805]) did think that their child would no longer get malaria as a result of having received IPTi. There were differences between the sites in this regard, with 27% (56/206) of mothers in Ghana thinking that their child would be completely protected against malaria, compared to only 3% (5/194) of mothers in Malawi. In Tanzania and Kenya the proportions were 15% (31/208) and 17% (34/197) respectively.

Some mothers (11% [91/805]) reported seeking less treatment for febrile illness in their infants while they were receiving IPTi compared to prior to IPTi. This varies from 2% in Malawi to 17% in Kenya and Tanzania). There is evidence that some of this reduction was due to exaggerated assumptions by mothers about the extent of protection offered by IPTi. However, the reduction is potentially due to less (perceived) illness after IPTi. Two percent (15/805) (all in Ghana) of mothers reported that they would be less likely to seek treatment or would delay seeking treatment because of IPTi.

In Ghana and Malawi a majority of mothers reported no change in bed net use during IPTi: 83% (170/206) and 87% (77/106) respectively. In Kenya and Tanzania mothers reported an increase in bed net use since IPTi: 25% (47/192) and 22% (23/106) respectively of mothers reported using bed nets more than prior to IPTi. This was largely due the fact that the two trials distributed nets to participants. For the 4% (26/612) of participants across these four sites who reported using bed nets less than before IPTi, the main reason was the net being damaged, followed by lack of mosquitoes. Only two participants directly related reduced net use to IPTi. In the Gabon study site there was 80% bed net use, of which 5% were insecticide-treated. However, there are no data on changes in use due to the trial.

## Discussion

The studies reported in this paper were part of a broad programme of research aimed at exploring the reception of IPTi in a wide range of settings using different drugs and regimens. In particular, the studies aimed to understand (potential) facilitating factors and barriers to longer-term implementation. While the investigation of an intervention in the context of a clinical trial is not ideal, it is often the only way of collecting acceptability data early enough to be able to make recommendations for improving access and long-term adherence. This project had the advantage of also being able to collect data from two implementation settings. These data, together with the data relating to anti-malarial drugs other than SP and to different regimens, combined with the results from the two earlier studies – one of which was also an implementation study, have generated a broad and generalizable set of data relating to the acceptability of IPTi across a broad range of settings.

A number of key factors have emerged relating to the acceptability of IPTi. They mainly relate to various aspects of local culture (both inside and outside the clinic), to the fact that IPTi is delivered through EPI, and to the nature of the different drugs and regimens (and how these are interpreted). There is a great deal of similarity regarding all these issues across the different sites.

In all the study communities, there was a general concern about infant health. Mothers were generally motivated to comply with prevention practices, whether traditional or biomedical, regardless of whether they fully understood them. Because non-compliance could be interpreted as neglect, there was also strong social pressure on mothers to comply and be seen as a "good mother". Traditional prevention practices and the belief that these are only partially effective were common at all sites, and the resonance between such traditional practices and EPI/IPTi, together with the general concern about infant health, formed a propitious context for the acceptance and routinization of both EPI and IPTi. These findings are similar to those from the earlier studies of IPTi reception in Mozambique and southern Tanzania [11,12].

Beyond local health culture, this acceptance was also related to a hierarchical culture of subservience and respect for authority that is particularly strong in the clinic setting where it is reinforced by dependence. As a result, women often said that they attended the clinic because they had been told to, or because they had to, or because it was "the law". In this context, women seemed to accept intimidation, and even occasionally overt bullying, without resisting or questioning. However, this sometimes had the opposite effect when fear of intimidation led to women avoiding the clinic. So although the culture of subservience works in both directions, its main influence is toward adherence. The relationship between mothers and health workers tends to be ambivalent: health workers have power and authority and women fear them, but women also respect and trust them, and this in turn contributes positively to clinic attendance and adherence. Their relationship is also reciprocal, and adherence can be seen as part of an exchange relationship between patients and health workers, with approval and assurance of future assistance being exchanged for adherence. The dynamics of this process are somewhat different in research settings compared to routine intervention settings. In the former the stakes are higher: there are more perceived risks and uncertainties (potentially dangerous drugs, blood stealing, being a research "guinea pig"), but the benefits are also potentially greater (more effective drugs, free and privileged access to health care) [19].

The social dynamics between health workers and women played an important role in the acceptance of and adherence to IPTi, but so too did the social dynamics amongst the women themselves. Vaccination clinics were also social events to which many women saw as an opportunity to dress up and get away from the daily routine of farm work and household chores, and socialize. This aspect attracted women to the clinic and thus contributed to attendance and adherence, but, as with the relationship with the health workers, there was an ambivalent aspect, and disapproval could lead to non-adherence. This is particularly worrying in Gabon, where not only being poorly dressed, but also having an unhealthy child could be a cause of peer disapproval and clinic avoidance.

Fathers tend not to play a major role in routine infant care (including health seeking and treatment), which is seen as the mother's job. However, when men perceive health-related issues as non-routine (for example in the case of research or the introduction of new drugs), they become more interested and may intervene (negatively) by forbidding participation or demanding withdrawal. This response seems to be based largely on lack of knowledge about the intervention or misinterpretations based on rumour. This was very clear in the study in Mozambique, in which almost all the women who refused to enrol in, or who defaulted from, the IPTi trial did so because of pressure from husbands who had heard rumours that the researchers stole blood [12]. This suggests that sensitization about new interventions or research should be delivered more broadly and specifically include men, even if the intervention or research focuses on women or children.

EPI is generally well accepted and has become routinized because it is perceived as beneficial and because it fits in to local health culture. In this context the addition of IPTi in the form of a single dose of a familiar drug tends to be accepted more-or-less without question (unless negative attention is focused on the intervention, for example in the form of rumours about skin peeling off or blood stealing). Moreover, women were already familiar with IPTp, which involves the use of the same drug for the same purpose (partial protection against malaria through the administration of an anti-malarial).

One of the main determinants of acceptability is whether people perceive the intervention as doing what they think it is supposed to do, and whether the benefits are perceived to outweigh the disadvantages. In the case of IPTi, much of this relates to the perceived characteristics of the anti-malarial drugs. As with the other factors discussed above, most of the factors relating to the characteristics of the drugs or the way they were administered had both facilitating and impeding potential. People balance the pros and cons of the medication and are willing to tolerate perceived disadvantages as long as the benefits are thought to be worthwhile. So, for example, mothers were prepared to accept some fever and local swelling in order to reap the health benefits of vaccination. Similarly, in the research sites in Kenya and Tanzania they appeared to be willing to accept the vomiting and fever that were reported to follow IPTi as a price worth paying for a more effective drug and less malaria (just as they were willing to take the risk of blood stealing in order to benefit from the free health care provided by the trial clinics). Because these influences are often impossible to separate, especially in the trial settings, it is difficult to be clear about what precisely motivates mothers to attend clinic and adhere to treatment, and this makes it difficult to assess how they would respond in a more routine implementation setting. Much will depend on how patients link specific characteristics to particular drugs. For example, in Tanzania the trialists linked the vomiting reported by mothers to mefloquine and chlorproguanil-dapsone (and not to SP and placebo). As mothers did not know which drug their child was on, they could only link it to the "more powerful" drugs. In an implementation setting, a clear perceived link between a particular drug and an unacceptable side effect might lead to widespread non-adherence, especially if other drugs that are perceived to be equally effective are available.

Many study participants liked SP: they were familiar with it, they had used it for years, and they knew it was generally safe, in spite of rumours at some sites about it causing people's skin to peel off. There were those who were convinced that it was the best drug for them personally. But there was also ambivalence due to the fact that on the one hand it has been replaced as first-line treatment, suggesting that it is no longer effective, and on the other it is being promoted for IPTp and IPTi. There were also those who did not like SP because they perceived it as ineffective. This may be because of resistance, but it could also be related to the fact that SP has a relatively slow onset of action and no antipyretic effect. It is therefore possible that, although some people are still satisfied with SP (and it is still generally acceptable for IPTp and IPTi), this may change as people become aware of more powerful drugs and start to expect them for both treatment and prevention. It is also possible that this interpretation will affect perceptions of existing first-line treatment in settings in which other drugs are being tested, because participants tend to assume that the trial drugs are more effective than those in routine use, and that new drugs are being tested because existing ones are becoming less effective.

It is clear from the data that the crushing of tablets and their administration to the child using cups and spoons is not popular: mothers thought it was unhygienic, health workers claimed it was time-consuming and observation confirmed that it was often unpractical. There was general agreement among both mothers and health staff that easily deliverable infant formulation would be necessary for longer-term acceptability. However, this does need to be interpreted in the context of the balancing of pros and cons mentioned above: women are willing to tolerate the messy administration of the drug (just as they are willing to accept some side effects) if they perceive it as increasing the health of their infant. In the case of health workers, acceptance may be achieved through the gradual routinization of IPTi into the EPI process. There are probably limits to this, however, and the continued addition of new interventions to EPI would probably need to be accompanied by some increase in (perceived) rewards.

The number of doses and the complexity of the regimen are also key factors in acceptability and adherence. It seems intuitively obvious that a single dose of a familiar drug administered under supervision in the clinic will achieve higher adherence than a multi-dose regimen of a new drug administered at home, and this is what the evidence suggests. This is strengthened by various other factors.

First, people may be less inclined to take three tablets if they can achieve what they want with only one – they then have some to spare for another occasion. Also, the fact that the new drugs being tested in the trials in Tanzania and Kenya were seen as more powerful supports the logic of saving some for later (why give three doses of a strong drug to achieve the same as you would achieve with a single dose of a weaker drug?). Multiple doses given in the clinic may be more feasible than multiple doses at home, but getting busy women to come for follow-up visits is likely to be a challenge.

Second, the mere fact of multiple doses taken at home increases the risk of forgetting, losing tablets, sharing with friends, etc. In the case of the Tanzania and Kenya trials, adherence was built into the trial design with compliance monitors and home visits (though the trials were fairly different in this regard, with monitors just assessing compliance in Tanzania, while Kenya staff supervised the actual taking of the drugs at home). Health workers and monitors agreed that that multiple doses at home would not work, and some mothers admitted that it would be challenging and that they would be inclined to forget or save doses for later. This could not be observed first hand in the trial sites, as the acceptability studies started at the end of implementation, but there is some evidence of this behaviour from the implementation study in southern Tanzania, where busy clinic staff sometimes gave mothers the single dose of SP to take at home, and home visits by the researchers revealed that the drug had not been administered [11,20].

However, there are reasons not to be entirely pessimistic about this. First, some women were conscientious and did adhere to medication, for example some of the women who were given SP for home administration did so properly; they understood the importance of taking the drug as prescribed and it is perhaps patronising to assume that uneducated rural women are completely incapable of understanding the importance of adherence [20]. Moreover, health workers in some of these settings do tend to be pessimistic about the ability of rural women to understand and comply with treatment [21]. Second, some health workers were nonetheless optimistic that women could be adherent to multiple doses if they were given proper information and the right degree of support. The data suggest that too much support (as in the intensive follow-up during the trials) perhaps leads to passivity and over-reliance on reminders and that in the longer term a more empowering approach might work better.

One of the major concerns relating to the introduction of IPTi into the EPI scheme was that if communities viewed IPTi negatively then this might lead to reduced EPI clinic attendance. There is little evidence, either from the data described in this paper or the studies in southern Tanzania and Mozambique, that IPTi in itself had any negative impact on attitudes to EPI or that it had any effect on EPI adherence. The few cases in which participants claimed that IPTi was a reason for avoiding EPI were largely related to the research process and the taking of blood in particular, rather than to IPTi per se.

Another concern was that the concurrent delivery of IPTi and immunization might lead people to perceive IPTi as immunization against malaria and as a result they might stop using other preventive measures such as bed nets. Mothers did not generally think that having received IPTi meant that their child would no longer get malaria because they tended to view all prevention practices as partial – attenuating rather than completely preventing disease. Moreover, even if women did think that their child was now immune to malaria, this would not necessarily mean that they would fail to seek treatment if the child became febrile because, due to the general difficulty in distinguishing between malaria and non-malarial fever (as shown by the fact that there is only a single term in most languages), mothers would be likely to seek treatment anyway.

## Conclusion

This paper has reported on a comprehensive study of the acceptability and reception IPTi in five settings in Africa. It also refers to already published studies in two additional settings, carried out by the same group. This programme of anthropological research has revealed a great deal of similarity across very different sites and settings and therefore allows some generalization relating to the implementation of IPTi across Africa. First, IPTi delivered through EPI fits well with local health cultures and appears to become easily routinized. It resonates with traditional practices (which should be evaluated for possible harmful effects), which appear to be widespread. As a result, deliberately fitting interventions more closely to local culture (by involving traditional healers or making use of the social culture of the clinic) could improve adherence. Second, there is little evidence that IPTi has any negative impact on attitudes to EPI or EPI adherence. There is also little evidence that the concurrent delivery of IPTi and immunization has led to people perceiving IPTi as immunization and changing existing health-seeking behaviour detrimentally. Third, local understanding of new drugs and regimens (and existing ones) is patchy and often inaccurate. Information provision should focus on issues that participants consider relevant (efficacy of different drugs, reasons for new drugs and regimens, and details of side effects and their meaning) and be broadened to include fathers. Informal information channels (such as those through which rumours spread) should be investigated further as a potential means of countering misinformation and reaching the wider community. Fourth, it is important that an infant formulation is developed, and this should ideally be a single dose, administered in the clinic as part of EPI.

## Competing interests

The authors declare that they have no competing interests.

## Authors' contributions

MG helped to conceive and design the study, managed the research programme, participated in data analysis and drafted the manuscript. CP supervised data collection in Kenya, managed the NVivo project for all sites, coded all interviews, analysed the quantitative data and contributed to drafting the manuscript. DPM supervised data collection in Malawi and commented on the draft manuscript. PA supervised data collection in Ghana and commented on the draft manuscript. FO and MJH supervised data collection in Kenya and commented on the draft manuscript. RG contributed to the design of the study, supervised data collection in Tanzania and commented on the draft manuscript. PA managed the field workers in Ghana and commented on the draft manuscript. RM and PM managed the field workers in Tanzania and commented on the draft manuscript. GO collected data in Kenya and commented on the draft manuscript. LS, RDN, PGK and MPG contributed to the design of the study and commented on the draft manuscript. RP conceived and designed the study, obtained project funding, managed the research programme, participated in data analysis and contributed to writing the paper. All authors read and approved the final manuscript.

## References

[B1] Egan A, Crawley J, Schellenberg D (2005). Intermittent preventive treatment for malaria control in infants: moving towards evidence-based policy and public health action. Trop Med Int Health.

[B2] Schellenberg D, Menendez C, Kahigwa E, Aponte J, Vidal J, Tanner M, Mshinda H, Alonso P (2001). Intermittent treatment for malaria and anaemia control at time of routine vaccinations in Tanzanian infants: a randomised, placebo-controlled trial. Lancet.

[B3] Chandramohan D, Owusu-Agyei S, Carneiro I, Awine T, Amponsa-Achiano K, Mensah N, Jaffar S, Baiden R, Hodgson A, Binka F, Greenwood B (2005). Cluster randomised trial of intermittent preventive treatment for malaria in infants in area of high, seasonal transmission in Ghana. BMJ.

[B4] Macete E, Aide P, Aponte JJ, Sanz S, Mandomando I, Espasa M, Sigauque B, Dobano C, Mabunda S, DgeDge M, Alonso P, Menendez C (2006). Intermittent preventive treatment for malaria control administered at the time of routine vaccinations in Mozambican infants: a randomized, placebo-controlled trial. J Infect Dis.

[B5] Kobbe R, Kreuzberg C, Adjei S, Thompson B, Langefeld I, Thompson PA, Abruquah HH, Kreuels B, Ayim M, Busch W, Marks F, Amoah K, Opoku E, Meyer CG, Adjei O, May J (2007). A randomized controlled trial of extended intermittent preventive antimalarial treatment in infants. Clin Infect Dis.

[B6] Mockenhaupt FP, Reither K, Zanger P, Roepcke F, Danquah I, Saad E, Ziniel P, Dzisi SY, Frempong M, Agana-Nsiire P, Amoo-Sakyi F, Otchwemah R, Cramer JP, Anemana SD, Dietz E, Bienzle U (2007). Intermittent preventive treatment in infants as a means of malaria control: a randomized, double-blind, and placebo-controlled trial in northern Ghana. Antimicrob Agents Chemother.

[B7] Grobusch MP, Lell B, Schwarz NG, Gabor J, Dornemann J, Potschke M, Oyakhirome S, Kiessling GC, Necek M, Langin MU, Klouwenberg PK, Klopfer A, Naumann B, Altun H, Agnandji ST, Goesch J, Decker M, Salazar CL, Supan C, Kombila DU, Borchert L, Koster KB, Pongratz P, Adegnika AA, Glasenapp I, Issifou S, Kremsner PG (2007). Intermittent preventive treatment against malaria in infants in Gabon-a randomized, double-blind, placebo-controlled trial. J Infect Dis.

[B8] Aponte JJ, Schellenberg D, Egan A, Breckenridge A, Carneiro I, Critchley J, Danquah I, Dodoo A, Kobbe R, Lell B, May J, Premji Z, Sanz S, Sevene E, Soulaymani-Becheikh R, Winstanley P, Adjei S, Anemana S, Chandramohan D, Issifou S, Mockenhaupt F, Owusu-Agyei S, Greenwood B, Grobusch MP, G KP, Macete E, Mshinda H, Newman RD, Slutsker L, Tanner M, Alonso P, Menendez C (2009). Intermittent Preventive Treatment for malaria control in African infants: Pooled analysis of safety and efficacy in six randomised controlled trials. Lancet.

[B9] Committee on the Perspectives on the Role of Intermittent Preventive Treatment for Malaria in Infants (2008). Assessment of the Role of Intermittent Preventive Treatment for Malaria in Infants: Letter Report.

[B10] IPTi Consortium. http://www.ipti-malaria.org.

[B11] Pool R, Mushi A, Schellenberg J, Mrisho M, Alonso P, Montgomery C, Tanner M, Mshinda H, Schellenberg D (2008). The acceptability of intermittent preventive treatment of malaria in infants (IPTi) delivered through the expanded programme of immunization in southern Tanzania. Malar J.

[B12] Pool R, Munguambe K, Macete E, Aide P, Juma G, Alonso P, Menendez C (2006). Community response to Intermittent Preventive Treatment delivered to infants (IPTi) through the EPI system in Manhiça, Mozambique. Trop Med Int Health.

[B13] Schellenberg JA, Mrisho M, Manzi F, Shirima K, Mbuya C, Mushi A, Ketende SC, Alonso P, Mshinda H, Tanner M, Schellenberg D (2008). Health and survival of young children in southern Tanzania. BMC Public Health.

[B14] Glaser B, Strauss A (1967). The Discovery of Grounded Theory: Strategies for Qualitative Research.

[B15] Strauss AL, Corbin J (1997). Grounded Theory in Practice.

[B16] Pool R, Kamali A, Whitworth JA (2006). Understanding sexual behaviour change in rural southwest Uganda: a multi-method study. AIDS Care.

[B17] Pool R (2008). Some issues arising from the use of qualitative methods in clinical trials. 2007 Royal Dutch Academy of Sciences (KNAW) Colloqium: Advising on Research Methods: 2008.

[B18] Schwarz NG, Gysels M, Pell C, Gabor J, Schlie M, Issifou S, Lell B, Kremsner PG, Grobusch MP, Pool R (2009). Reasons for Non-Adherence to Vaccination at Mother and Child Care Clinics (MCC) in Lambaréné/Gabon. Vaccine.

[B19] Geissler PW, Kelly A, Imoukhuede B, Pool R (2008). 'He is now like a brother, I can even give him some blood' – Relational ethics and material exchanges in a malaria vaccine trial community' in The Gambia. Soc Sci Med.

[B20] Mushi AK (2008). Reaching the poorest children in rural southern Tanzania: socio cultural perspectives for delivery and uptake of preventive child health interventions.

[B21] Montgomery CM, Mwengee W, Kong'ong'o M, Pool R (2006). 'To help them is to educate them': power and pedagogy in the prevention and treatment of malaria in Tanzania'. Trop Med Int Health.

